# Sacral Spinal Cord Transection and Isolated Sacral Cord Preparation to Study Chronic Spinal Cord Injury in Adult Mice

**DOI:** 10.21769/BioProtoc.2784

**Published:** 2018-04-05

**Authors:** Carmelo Bellardita, Maite Marcantoni, Peter Löw, Ole Kiehn

**Affiliations:** 1 Department of Neuroscience, University of Copenhagen, Copenhagen, Denmark; 2 Department of Neuroscience, Karolinska Institutet, Stockholm, Sweden

**Keywords:** Spinal cord injury, Complete transection, *in vitro* preparation, Sacral spinal cord, Spasticity

## Abstract

Spinal cord injury (SCI) is characterized by multiple sensory/motor impairments that arise from different underlying neural mechanisms. Linking specific sensory/motor impairments to neural mechanism is limited by a lack of direct experimental access to these neural circuits. Here, we describe an experimental model which addresses this shortcoming. We generated a mouse model of chronic spinal cord injury that reliably reproduces spasticity observed after SCI, while at the same time allows study of motor impairments *in vivo* and in an *in vitro* preparation of the spinal cord. The model allows for the combination of mouse genetics in *in vitro* and *in vivo* conditions with advanced imaging, behavioral analysis, and detailed electrophysiology, techniques which are not easily applied in conventional SCI models.

## Background

Spinal cord injury results in devastating sensory-motor disabilities. Extensive work in animal models has investigated the pathophysiological state of spinal circuits after SCI. Most studies in spinal cord injury are carried out in animal models relevant for clinical evaluation of motor impairment and recovery after SCI (Sharif-Alhoseini *et al*., 2017). One of the main limitations of these models is the difficulty to relate clinical features of sensory-motor dysfunction to specific cellular mechanism(s). In the last decade, new insights into possible cellular mechanisms underlying motor impairments after SCI have come from transection models of SCI. Here, the sacral spinal cord is surgically transected resulting in paralysis only of the tail muscles. This model was first introduced in cats (Ritz *et al*., 1992), and later in rats (Bennett *et al*., 1999), and has provided insights into cellular mechanisms underlying sensory-motor dysfunction after injury (Bennett *et al*., 2001). Nevertheless, the genetic tools for manipulating neuronal activity in cats and rats are very limited. In contrast, mice allow electrophysiology to be combined with genetics for identification and manipulation of the activity of specific neurons in the spinal cord (Jiang and Heckman, 2006; Kiehn, 2016). In recent efforts, these advantages have allowed optical approaches (*e.g.*, optogenetics, calcium imaging of defined neuronal subtypes) to be used for dissection of the neural mechanisms underlying muscle spasms after SCI (Bellardita *et al*., 2017). In this protocol, we describe the technical aspects of sacral spinal cord transection in adult mice, and the subsequent use of *in vitro* sacral spinal cord preparations for direct examination of the neural mechanisms which cause spasm in chronic spinal cord injury.

## Materials and Reagents

1. Glass micropipettes (Harvard Apparatus, catalog number: GC150F-10)
2. Wood stick cotton tip swabs (Medline Scientific, catalog number: 300230S)
3. Non-sterile gauze swabs (Kruuse, catalog number: 160120)
4. Surgery cover 60 x 90 cm (Kruuse, catalog number: 141770)
5. Absorbent swabs (Kettenbach, catalog number: 001911)
6. Suture, straight cutting needles, non-absorbable (eSutures, Ethicon, catalog number: K889H)
7. Hypodermic needles 26 G brown 16 mm (BD, Microlance, catalog number: 304300)
8. 23-Gauge needle (PrecisionGlide IM; BD, catalog number: 305145)
9. Vetbond tissue adhesive (3M, catalog number: 1469C)
10. Surgical glue (3M, Vetbond, catalog number: 372146)
11. Facial Tissue (VWR, catalog number: 115-0600)
12. Bench liner paper (ScienceWare, VWR, catalog number: 470145-292)
13. 8 weeks old mice (JAX Mice Strain; THE JACKSON LABORATORY, catalog number: 000664)
  *Note: All experiments should be performed in accordance with relevant guidelines and regulations. The local Swedish and Danish ethical committees approved all procedures described here*.
14. 100% oxygen (O_2_)
15. Isoflurane (Baxter, catalog number: 1001936060)
16. Lidocaine Hydrochloride (Sigma-Aldrich, catalog number: BP214)
17. Betadine^®^ Surgical Scrub (povidone-iodine, 7.5%)
18. Xilocaine (1%)
19. Buprenorphine hydrochloride (Reckitt Benckiser Healthcare, 0.3 mg/ml)
20. Carprofen (Canidryl, catalog number: 122964)
21. Physiologic solution (0.9% sodium chloride) (Grovet, Braun Ecotainer, catalog number: 99512)
22. Viscotears liquid gel for dry eyes (Novartis, catalog number: 2082642)
23. 70% ethanol
24. Sylgard (Sigma-Aldrich, catalog number: 761028-5EA)
25. Sodium chloride (NaCl, Sigma-Aldrich, catalog number: 433209)
  *Note: This product has been discontinued*.
26. Potassium chloride (KCl, Sigma-Aldrich, catalog number: P9541)
27. Glucose (Sigma-Aldrich, catalog number: G8270)
28. Sodium bicarbonate (NaHCO_3_, Sigma-Aldrich, catalog number: S5761)
29. Magnesium sulfate heptahydrate (MgSO_4_·7H_2_O, Sigma-Aldrich, catalog number: 63138)
30. Potassium phosphate monobasic (KH_2_PO_4_, Sigma-Aldrich, catalog number: 92214)
31. Calcium chloride (CaCl_2_, Sigma-Aldrich, catalog number: 449709)
32. Magnesium chloride (MgCl_2_, Sigma-Aldrich, catalog number: V000149)
33. HEPES (Sigma-Aldrich, catalog number: H3375)
34. Ringer’s solution (see Recipes)
35. Oxygenated modified artificial cerebrospinal fluid (mACSF, see Recipes)

## Equipment

1. Forceps (Fine Science Tools, catalog number: 11251-10)
2. Toothed forceps (Fine Science Tools, catalog number: 11154-10)
3. Vannas Spring scissors–Micro-serrated (Fine Science Tools, catalog number: 15007-08)
4. Dumont No. 2 laminectomy forceps (Fine Science Tools, catalog number: 11223-20)
5. Fine Scissors–Tungsten Carbide (Fine Science Tools, catalog number: 14568-09)
6. Fine Scissors–Tungsten Carbide & ToughCut (Fine Science Tool, catalog number: 14558-11)
7. Rechargeable animal clipper (Wahl Arco)
8. Scalpel (Fine Science Tools, catalog number: 10020-00)
9. Temperature-controlled variable heat pad (K&H Manufacturing, model: 1009)
10. Diaphragm vacuum pump-lubricated-single-stage (Environmental Express, catalog number: EE0753280)
11. Isofluorane vaporizer (Soarmed, model: MSS-3)

## Procedure

**Figure 1:**
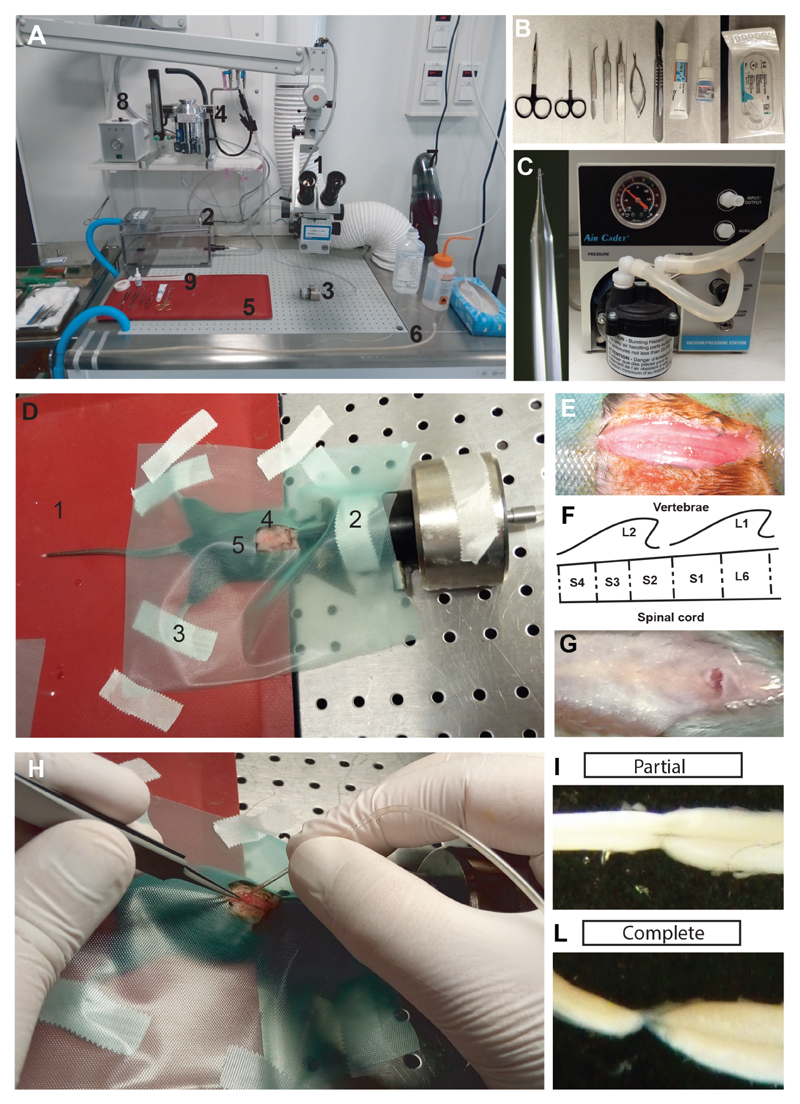
Lesion of the sacral spinal cord in adult mice. A. Area prepared for the surgery with easy access to the necessary equipment: 1) Dissection microscope; 2) Induction chamber for anesthesia; 3) Facial mask for anesthesia; 4) Isofluorane vaporizer; 5) Heating pad; 6) Glass pipette for aspirating the spinal cord connected to the vacuum pump; 7) Vacuum cleaner; 8) Device for surgical instrument sterilization. B. Surgical instruments for lesioning the sacral spinal cord with (from left to right): scissors, forceps, eye ointment, veterinary glue, and suture. C. Glass pipette and vacuum pump for aspirating the spinal cord. D. A mouse prepped for the lesioning procedure. 1) Heating pad; 2) Facial mask to deliver anesthesia; 3) Strips of tape for preventing sudden movements; 4) Area of interest shaved and prepped with sodium iodine; 5) Green cover for the mouse body with a work window in the area of interest. E. Surgical incision of the skin in the area of interest. F. Schematic of the surgery area with the vertebral body L1 and L2 with the second sacral segment of the spinal cord. G. Incision at the level of the L2 vertebral body after cutting the ligamentum flavum. H. Aspiration of the spinal cord using the glass pipette. I-L. Examples of dissected, lesioned sacral cords two months after SCI with either an incomplete (I) or complete (L) lesion.

**Figure 2:**
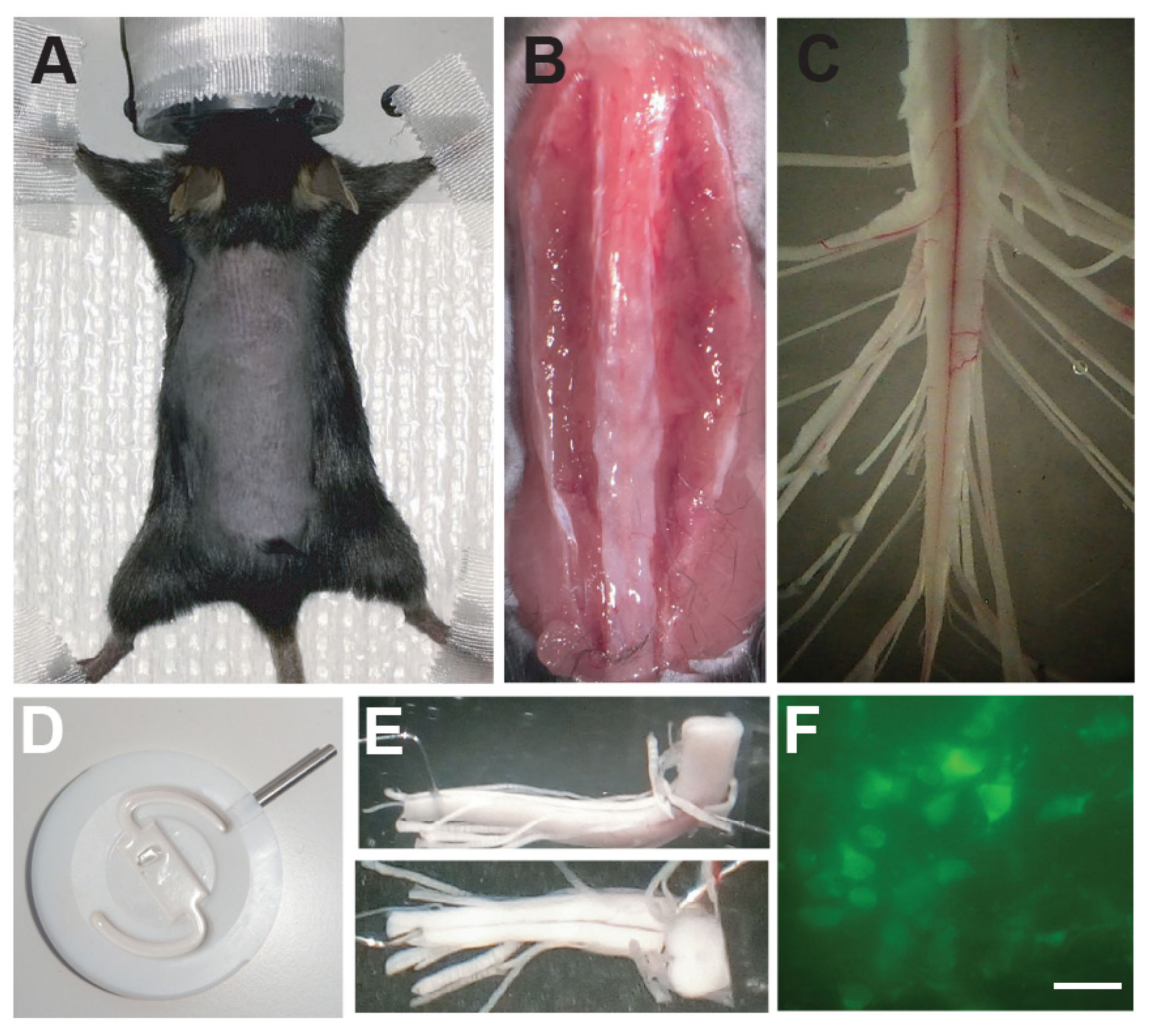
Dissection of the sacral spinal cord from adult mouse. A. Adult lesioned mouse under anesthesia and prepped for dissection of the sacral spinal cord. B. Incision of the skin and isolation of the spinal column from muscles and tendons. C. Isolated sacral spinal cord after dissection. D. Recording chamber for simultaneous calcium imaging of spinal interneurons and recording of motor activity. E. Sacral spinal cord positioned in the recording chamber with the transverse cut facing the microscope. F. Example of spinal neurons from a spinal cord of a *Vglut2^Cre^* (Borgius *et al*., 2010):: Rosa26-LSL-GCaMP3 (Ai38) mouse during calcium imaging. Scale bar = 20 μm.

- Complete lesion of the sacral spinal cord
  1. Prepare a clean and disinfected area dedicated to rodent surgery with only the equipment related to surgery (Figure 1A). Place the surgical instruments in an area which can be easily accessed during the procedure (Figure 1B).
  2. Prepare a glass pipette with a diameter of 50-100 μm from a glass capillary (Figure 1C). The glass capillary is pulled, and the tip is then manually adapted to the spinal cord. Connect the glass pipette to a vacuum pump through a series of silicon tubes of increasing diameter. The pulled glass pipette will be used in the surgery for suction-transection of the spinal cord.
  3. Weigh the mouse before surgery. Monitor the weight of the animal for the next 15 days to evaluate for the potential loss of weight after surgery.
  4. Place the mouse in a sealed induction chamber with 5% isoflurane/95% oxygen until it is deeply anesthetized (Figure 1A.2).
  5. Move the mouse from the induction chamber to the area dedicated to the surgery and prepare it for the surgery (Figure 1D):
    - Position the mouse ventral side down on a heating pad to maintain body temperature (37 °C) constant during the entire procedure (Figure 1D.1).
    - Use 2% isofluorane for the entire period of the surgery. Deliver isofluorane to the mouse through a facial mask (Figure 1D.2).
    - Check reflexes of the animal to verify an appropriate state of anesthesia. There should be no pinch-evoked reflexes. We assessed the pedal withdrawal reflex by pinching the tail and the metacarpal region of the hind foot.
    - Secure the animal to make sure it will not move during the surgery (as might be caused by touching the dorsal roots). Secure the animal with strips of tape attached to the limbs. Avoid excessive stretching of the limbs, which may damage joints as well as impair the animal’s breathing (Figure 1D.3).
    - Shave the back of the mouse along the rostrocaudal axis of the spinal column. Apply sodium iodine to the shaved area and leave it for 5 min (Figure 1D.4). Apply eye ointment to protect the eyes during surgery.
    - Remember to avoid resting your hands or instruments on the mouse thorax. The external pressure may interfere with respiration and/or blood circulation.
    - Apply a surgical cover to the body of the mouse, leaving a window at the point of incision (Figure 1D.5).
  6. Localization of the second sacral segment and transection:
    - Use two fingers to localize the T12 vertebral body. The T12 vertebra has the longest spinous process of all vertebrae, and if the spine of the mouse is put into flexion, the T12 vertebra protrudes outward in the spinal column. With a scalpel, make a longitudinal incision of the skin from approximately the T12 to the L4 vertebral bodies (Figure 1E).
    - The second sacral segment (S2) of the spinal cord lies beneath the rostral part of L2 vertebral body, on the boundaries between the L1 and L2 vertebral body. The spinous process of L2 points rostrally, and should be used as a landmark for making a deep vertical incision with a small eye scissor. If the cut is performed vertically, it will reveal the ligamentum flavum between the L1 and L2 vertebral bodies (Figure 1F). For a better understanding of the anatomical landmarks, and especially the relationship between lumbar vertebral bodies and the spinal cord in adult mice, refer to Harrison *et al.*, 2013.
    - Cut the ligamentum flavum with the eye scissor. The spinal cord will appear with a dorsal artery lying in the midline (Figure 1G).
    - Apply Xilocaine (1%) on the top of the cord to prevent movements elicited by touching the spinal cord or the dorsal roots. Wait for the drug to take effect (about half a minute) and then use fine forceps to position the dorsal roots as lateral as possible. In the case of other structural damage (*e.g.*, bones, arteries, tendons), the surgeon should consider discluding the animal from subsequent analysis.
    - If the cut to the ligamentum flavum is performed correctly, no other damage will be caused to the surrounding tissue (muscles, ligament or skin), and no blood should be visible.
    - Starting on one side of the cord, use the glass pipette attached to vacuum suction to remove the S2 spinal tissue. Keep aspirating tissue until a complete discontinuity is observed between the rostral and the caudal ends of the cord, in total corresponding to one segment.
  7. Suture the surgical wound and let the animal recover:
    - Suture the muscle around the spinal column at the injury site to protect the cord, and use veterinary glue close the skin.
    - Give post-surgery treatment of Buprenorphine (0.1 mg/kg), Carprofen (5 mg/kg) and, if necessary, 0.3 ml of sterile physiologic solution subcutaneously for 2 to 5 days post-surgery.
    - Turn the anesthesia off and place the mouse back in the cage with a heating pad to keep the animal warm for a period of 1-2 h. The animal may be housed alone for the first week, and thereafter, if the recovery is complete, it may be housed with another animal. Special cage bedding is not necessary.
    - Monitor the animal daily for signs of distress, including weight loss (a > 10% drop in body weight should be avoided), dehydration, or infection. In any of these cases, the surgeon should consult with a veterinarian for suggestions and solutions.
    - The injury should only cause paralysis of the tail muscles, and should not affect the bladder or hindlimbs. However, during daily postoperative care it is important to monitor for bladder dysfunction, which can sometimes occur if the injury site is too rostral.
    *Note: The limited visibility caused by a small working area during the surgery makes it difficult to reliably evaluate the completeness of the lesion. Therefore, all lesions should be evaluated visually after the end the experiment after dissection of the cord (Figures 1I-1L)*.
- Dissection of the sacral spinal cord of adult chronic spinalized mice
  1. Prepare a clean disinfected area dedicated to rodent surgery with easy access to the equipment necessary for isolation of the spinal cord (similar to that of Figure 1A).
  2. Place the animal in a chamber for induction of anesthesia (5% isofluorane/95% oxygen), and move the animal from the induction chamber to the dissection table when deeply anesthetized. Check reflexes as in Step A4c.
  3. Place the mouse on a bench liner paper (Scienceware) and apply isofluorane (2%) through the facial mask. Check the reflexes of an appropriate state of anesthesia as in Step A5c. Apply strips of tape to secure the limbs, shave the back, and clean the area with alcohol (Figure 2A).
    *Note: In this step, the animal does not have to be positioned on a heating pad as a lower temperature will decrease the metabolism, improving the dissection of the cord; eye ointment is not necessary since the procedure will last few minutes*.
  4. Laminectomy along the site of interest:
    - Identify T12 as described above.
    - Cut the skin from about the T12 vertebral body to L5 vertebral body. Keep in mind that the second sacral segment of the spinal cord is beneath the second vertebral body of the lumbar spinal cord (L2).
    - Expose the spinal column by cutting the muscles and tendons around it (Figure 2B).
    - Localize the spinous process of the T13 vertebral body (the T13 vertebra has the last pair of ribs attached), and start a dorsal laminectomy (a surgical procedure removing the dorsal portion of the vertebrae) in the rostro-caudal direction.
    - Begin to perfuse the spinal column with cold mACSF (20 ml/min) to slow down metabolism and reduce blood flow in the site of interest.
    - Proceed with the laminectomy by cutting the left and right sides of the vertebral body with the scissor.
    - Avoid damaging the spinal cord with the scissor, which can sometimes occur when moving the tip of the scissor from the left to right sides of the vertebrae (or vice versa). Damage may result in contusion or bruises of the spinal cord.
    - Use a continuous flow (20 ml/min) of cold (~4 °C), oxygenated mACSF on the spinal cord.
  5. Isolation of the sacral spinal cord:
    - When the spinal column is exposed form the caudal lumbar segments to the cauda equina, the laminectomy is complete (Figure 2C).
    - Give pure oxygen to the mouse through the facial mask for about 5 min to increase blood oxygenation levels.
    - Cut the skin at the level of the abdominal muscles and save the abdominal artery. This cut will cause a decrease in blood pressure, preventing an overflow of blood at the level of the cord during isolation.
    - Cut the cord at the level of the caudal lumbar segments and proceed cutting the ventral roots on the right and left sides of the cord to isolate it from the spinal column.
    - Pay special attention when you reach the site of the lesion. At the level of the injury, the dura mater is often attached to the vertebral body, requiring careful detachment of the spinal cord and dura from the rest of the spinal column.
    - Once the cord is completely detached, move it in a dissection chamber with a continuous flow (20 ml/min) of cold oxygenated-mACSF.
    - Carefully and completely remove the dura mater from the spinal cord to allow greater diffusion of oxygenation into the tissue. Cut the spinal cord, the dorsal root and the ventral roots to decrease the length and allow an easier recognition of the different segments and roots during the experiment (Figure 2C).
    - Once all roots of the cord are cut and the isolation of the cord is complete, the spinal cord can be moved to a perfusion chamber covered with Sylgard (Figure 2D). Provide a continuous flow of mACSF at room temperature (3-7 ml/min).
  6. *In vitro* preparation of the sacral spinal cord for simultaneous calcium imaging of spinal interneurons and ventral root recording:
    - Move the spinal cord to the recording chamber. The recording chamber has a Sylgard bottom and a Sylgard ‘bridge’ attached which allows the cord to be placed in an L-shaped position (Figure 2E), such that the coronal plane of the spinal cord can be imaged with an objective lens.
    - Keep a continuous flow of oxygenated Ringer solution for the duration of the experiment.
    - The cord is pinned down ventral side up from the most caudal end to ensure mechanical stability. The rostral end of the cord leans onto the bridge and a bended minute pin is used as a hook to keep the cord in place (Figure 2E).
    - Suction electrodes are used for recording motor activity (S4-Co1) and stimulating dorsal roots.
    - Calcium imaging is performed by lowering the objective over the region of interest (Figure 2F) once the glass suction electrodes are connected to the roots.
    *Notes*:
    - *The procedure from the Step B5 to the Step B6 should not take more than a minute otherwise the preparation’s viability may be compromised*.
    - *The dissection of the sacral spinal cord in lesioned mice (Procedure B) should be performed > 2 months after lesion for studying chronic spinal cord injury*.

## Data analysis

The effect of the injury, its reproducibility, and quantification of the electrophysiological and calcium imaging data are conceptualized and quantified in our study ‘Spatiotemporal correlation of spinal network dynamics underlying spasms in chronic spinalized mice’ (Bellardita *et al*., 2017).

## Notes

1. The quality of the lesion is largely dependent on the manual skills of the surgeon. These techniques require experience with identification of anatomical structures, and it can be common for newly trained surgeons to disturb the lumbar dorsal/ventral root–with negative consequence to the sensory/motor function of the hindlimbs.
2. During aspiration of the cord, the main dorsal artery should be left intact. Severing the dorsal artery during the procedure can result in degeneration of the spinal cord below the injury site. This effect may present behaviorally as flaccidity of the tail, and is typically referred to as dead tail syndrome.
3. For obtaining high quality recordings in the *in vitro* preparation, special care should be used in maintaining the temperature of the solution during the laminectomy close to 4 °C to reduce cellular death.

## Recipes

1. Ringer’s solution
  111 mM NaCl
  3 mM KCl
  11 mM glucose
  25 mM NaHCO_3_
  1.25 mM MgSO_4_
  1.1 mM KH_2_PO_4_
  2.5 mM CaCl_2_
  Oxygenated in 95% O_2_ and 5% CO_2_ to obtain a pH of 7.4 and maintained at 22-24 °C
2. Oxygenated modified artificial cerebrospinal fluid
  125 mM Choline-Cl
  1.9 mM KCl
  1 mM CaCl_2_
  7 mM MgCl_2_
  1.2 mM KH_2_PO_4_
  10 mM HEPES
  25 mM glucose
  *Note: Storing of the solutions is important. The solutions can be saved in refrigeration < 10 °C and must be transparent. In case of any concern about the possibility of contamination or bacterial growth, the solution should be replaced*.
